# The prevalence of hepatitis B virus infection in HIV-positive and HIV-negative infants: KwaZulu-Natal, South Africa

**DOI:** 10.4102/ajlm.v5i1.283

**Published:** 2016-03-31

**Authors:** Nokukhanya Mdlalose, Raveen Parboosing, Pravi Moodley

**Affiliations:** 1 Department of Virology, National Health Laboratory Service, University of KwaZulu-Natal, South Africa

## Abstract

**Background:**

The prevalence of hepatitis B virus (HBV) amongst South African infants and children has been reported in the pre-HIV era. Despite the reported high prevalence of HIV in the general population of South Africa, the rate of HIV/HBV co-infection amongst infants and children remains poorly reported.

**Objectives:**

We describe the prevalence of HBV infection amongst HIV-positive and HIV-negative infants by molecular methods of diagnosis using dried blood spot samples.

**Methods:**

This retrospective cross-sectional study was conducted between July 2011 and December 2011 in an academic referral laboratory offering viral diagnostic services to the entire KwaZulu-Natal province of South Africa. A total of 322 study samples were collected from discarded residual dried blood spot samples following routine infant diagnosis of HIV. Equal proportions of HIV-positive and HIV-negative infant specimens were studied. Statistical differences in the prevalence of HBV between the HIV-positive and HIV-negative samples were calculated using the Pearson chi-square test, and a *p*-value < 0.05 was considered statistically significant. Further testing for HBV DNA using a nested polymerase chain reaction method was performed.

**Results:**

The overall prevalence of HBV was 10%. In the HIV-positive group, 21 of 161 infants tested positive for HBV compared with 12 of 161 HIV-negative infants who tested positive for HBV. The proportion of infants infected with HBV was marginally higher amongst HIV-positive infants (13.0%; 95% CI 6.8–19.9) compared with HIV-negative infants (7.5%; 95% CI 2.5–13.7; *P* = 0.098), though not statistically significant.

**Conclusion:**

The finding of a 10% HBV prevalence in this infant cohort is clinically significant. The non-statistically significant difference in HBV prevalence between the HIV-positive and HIV-negative infants suggests that high prevalence of HBV infection in children may be a problem independent of HIV.

## Introduction

Hepatitis B virus (HBV) infection remains a major health care problem throughout the world. Of the two billion people infected with the virus, more than 350 million are chronic carriers of HBV, and approximately 600 000 die annually from HBV-related complications, including hepatocellular carcinoma.^1^ Expanded vaccination initiatives have been associated with a decline in global HBV prevalence, but national and sub-national prevalence data are still required to monitor disease burden continuously and guide vaccine policies.^2^

South Africa introduced compulsory infant vaccination against HBV as part of an expanded programme on immunisation in 1995.^3^ Pre-immunisation seroprevalence of HBV infection amongst children from the Eastern Cape province of South Africa was reported to be 10.4% in a study conducted between 1995 and 1996.^4^ A study conducted in the Limpopo province showed that the vaccination programme had a positive impact, and there was evidence of elimination of HBV surface antigen carriage amongst children aged 5 years or younger.^5^ However, another South African study suggested that the response to HBV vaccination amongst HIV-positive infants may be sub-optimal.^6^

There is a growing body of evidence showing that the prevalence of HBV is significantly higher amongst HIV-positive individuals, mainly because of shared risk factors.^7,8,9,10^ Despite the generally reported high prevalence of HIV and HBV, there is still no clear government policy in South Africa for screening or active case finding for HBV amongst high-risk individuals or pregnant women.^9^ The general prevalence of HBV amongst pregnant women is under-reported, with a retrospective cross-sectional study in the Western Cape province of South Africa reporting a regional seroprevalence of 3.4% amongst HIV-positive women and 2.9% amongst HIV-negative women.^11^ A study in the KwaZulu-Natal province of South Africa reported a 5.3% HBV prevalence and a 3.1% HIV/HBV co-infection prevalence amongst pregnant women in a province where antenatal HIV prevalence was reported to be 37.4% in 2012.^12^ The prevalence of HBV amongst South African infants and children was widely reported in the pre-HIV era. Vardas et al. reported the overall prevalence of HBV in the Eastern Cape to be 10.4%, ranging from 8.9% in the 0–6-months age group to 15.7% in the 61–72-months age group between 1995 and 1996.^4^ Another, earlier study in KwaZulu-Natal province reported the prevalence of HBV in urban, rural and institutionalised children to be 10%, 18.5% and 25.1%, respectively.^13^ In the era of the South African HIV epidemic, a study conducted in the Western Cape in 2011 found the prevalence of HBV amongst HIV-exposed infants to be 0.4%.^14^ The rate of HIV/HBV co-infection amongst infants and children remains poorly reported compared with reports of high exposure to HBV in HIV-infected pregnant women.^9^

The concept of using whole blood or plasma blotted onto a filter paper to screen for neonatal metabolic disorders was introduced in Scotland by Guthrie et al. in 1963.^15^ The use of dried blood spot (DBS) samples for the detection of HIV, as well as the stability of nucleic acids in this sample collection method, is well described.^16,17^ DBS samples have been shown not only to be reliable and convenient, but also to have improved access to laboratory testing in developing countries.^18^ The stability of markers for the testing of other infectious diseases, including HBV, on DBS samples is well documented.^19,20^

The objective of this study was to describe the prevalence of HBV infection amongst HIV-positive and HIV-negative infants (aged between 6 weeks and 18 months) tested for early-infant diagnosis of HIV in KwaZulu-Natal by detecting HBV DNA using polymerase chain reaction (PCR) in DBS samples. We also evaluated whether the prevalence of HBV in this population group was influenced by HIV co-infection.

## Research method and design

### Ethical considerations

Ethical approval was obtained from the Biomedical Research and Ethics Committee (BREC) of the University of KwaZulu-Natal (BE243/010). The samples were sequentially selected from those discarded and anonymised.

### Study population and design

This retrospective cross-sectional study was conducted in the Department of Virology at Inkosi Albert Luthuli Central Hospital, located in the city of Durban, KwaZulu-Natal, South Africa. The Department of Virology is an academic referral laboratory that offers diagnostic virology services to the entire KwaZulu-Natal province and is a laboratory of the National Health Laboratory Service. Approximately 9000 DBS samples are received by the laboratory per month for routine diagnostic detection of HIV using a commercial kit, namely the HIV DNA PCR COBAS^®^ Ampliprep/COBAS TaqMan^®^ HIV-1 qualitative test (Roche Molecular Systems, Inc., Branchburg, New Jersey, United States).

The study samples were randomly selected from residual samples scheduled to be discarded after testing for early-infant diagnosis of HIV. Samples with a full blood spot from infants aged 18 months or younger were included for HBV testing. Samples with inadequate blood spots or from patients aged older than 18 months were excluded. Once the HIV results and age were recorded, the samples were anonymised and given unique study numbers. The study samples were collected over a six-month period (July 2011 to December 2011). The samples were stored in individual plastic packets with desiccant at room temperature until tested for HBV using the nested PCR method.

The sample size for the number of HIV-positive and HIV-negative samples was calculated using an HIV prevalence of 29.3%, as reported in the South African Annual National Surveillance in Antenatal Clinics report of 2008.^12^ The sample size of 322 was based on an estimated 250 000 live births in KwaZulu-Natal^21^ and allowed for a confidence interval (CI) of 95% for a population epidemiological survey, as calculated using Epi Info™, version 3.5.1 (Centers for Disease Control and Prevention, Atlanta, Georgia, United States). Equal proportions of HIV-positive and HIV-negative infant specimens were included.

### Laboratory methods

A qualitative in-house nested PCR method^22^ was used for the detection of HBV DNA in the DBS samples. The primers (Table 1) targeted the surface gene of the HBV genome. The first outer set of primers was used to amplify the entire coding region for the large-surface antigen gene of the HBV genome, which incorporated the middle-surface antigen and the small-surface antigen. The nested primers amplified the preS2 region and most of the surface antigen. Details of this method have been published previously.^23^

**TABLE 1:** PCR primers used for detection of HBV DNA.

Primer	Sequence (5´–3´)	Amplicon size
First-round PCR primers	-	-
Asn 1018	5´-CCA CAT TGT GTA AAT GGA GCA GC-3´	1388-1427 bp
Sn 2833	5´-CTT GGG AAC AAG AGC TAC AGC AT-3´	-
Nested PCR primers	-	-
Asn 688	5´-ACG CCT ACG AAC CAC TGA ACA AAT-3´	780 bp
Sn 3130	5´-CCT CCT GCC TCC ACC AAT CG-3´	-

HBV, hepatitis B virus.

Nucleic acid extraction was achieved using the MagNA Pure LC™ DNA Isolation kit III on the Roche MagNA Pure™ LC instrument (Roche Molecular Diagnostics, Mannheim, Germany). A pre-extraction step was performed^24^ as a modification to the manufacturer’s protocol for DBS extraction. A whole-blood spot containing approximately 50 µL of dried blood was cut from the filter paper and added into a 300 µL mixture of lysis buffer with proteinase K and elution buffer in a 1-mL microtube. The tubes were incubated on a heating block for 10 min at 65 °C and another 10 min at 95 °C, then centrifuged at 1500 g for 3 minutes. A 200-µL lysate was then transferred for automated nucleic acid extraction.

Amplification was carried out using the GenAmp 9700™ thermocycler (Applied Biosystems™, Life Technologies, Carlsbad, California, United States). The master mix reagents for both PCR rounds were prepared in a separate clean laboratory and kept on an ice block to prevent nonspecific reactions and minimise the formation of primer dimers. The first-round cycling programme had 36 cycles and dilution of the first-round PCR products and addition to the nested master mix were performed in a biosafety cabinet to minimise the inherent contamination risk of the nested PCR method. Each run contained one negative and one positive control. Owing to the efficacy of the nested PCR, the first-round products were diluted in a 1:2 ratio to minimise background on the agarose gel and increase efficacy of the PCR. After a further 36 cycles of nested PCR, a fragment of approximately 780 bp was visualised using 1% agarose gel electrophoresis. A 100-bp DNA molecular weight marker XIV (Roche Molecular Diagnostics, Mannheim, Germany) was used to identify the desired amplicon size.

### Data analysis and statistical methods

Data were analysed using SPSS^®^, version 22 (IBM^®^ Inc., Armonk, New York, United States). Statistical differences in the prevalence of HBV between the HIV-positive and HIV-negative samples were calculated using the Pearson chi-square test. Further analysis of the influence of sex and age on the prevalence of HBV was conducted using stratified analysis. A *P-*value of < 0.05 was regarded as statistically significant. CIs were calculated by bootstrapping, using the default options in SPSS^®^.

## Results

Amongst the 161 samples from HIV-positive infants and 161 samples from HIV-negative infants, the overall prevalence of HBV was 10% (Table 2). Amongst the HIV-positive infants, 21 tested positive for HBV compared with 12 HIV-negative infants who tested positive for HBV. The proportion of HBV infection amongst HIV-positive infants was found to be marginally higher than amongst HIV-negative infants (13.0%, 95% CI: 6.8–19.9 for HIV-positive infants vs 7.5%, 95% CI: 2.5–13.7 for HIV-negative infants; *P* = 0.098). The observed difference in HBV infection between boys and girls (Table 3) was not found to be statistically significant (*P* = 0.33). Further analysis by age categories showed approximately equal proportions of HBV infection amongst all categories for infants aged 52 weeks or younger, with the exception that all samples from infants aged 53 weeks or older were negative for HBV (Table 4). The chi-squared test showed that there were no differences in HBV infection across all age categories (*P* = 0.73).

**TABLE 2:** Prevalence of HBV infection amongst HIV-positive and HIV-negative infants, KwaZulu-Natal, South Africa (July 2011 to December 2011).†

HBV infection	HIV-positive *N* (%)	HIV-negative *N* (%)	*P*-value	Odds ratio
Positive	21/161 (13.04)	12/161 (7.45)	0.098	1.86
Negative	140/161 (86.96)	149/161 (92.55)	-	(0.88–3.93)

HBV, hepatitis B virus.

† HBV infection was determined by PCR performed on leftover dried blood-spot samples from 322 infants referred to the Department of Virology at Inkosi Albert Luthuli Central Hospital, Durban, South Africa, for routine early-infant diagnosis of HIV. The study included an equal number of samples from HIV-positive and HIV-negative infants.

**TABLE 3:** HBV infection amongst HIV-positive and HIV-negative infants stratified by sex, KwaZulu-Natal, South Africa (July 2011 to December 2011).

Sex†	HBV-positive (%)	HBV-negative (%)
Boys	6 (6.4)	88 (93.6)
Girls	12 (11.2)	95 (88.8)
Unknown	15 (12.4)	106 (87.6)
**Total**	**33 (10.2)**	**289 (89.8)**

HBV, hepatitis B virus.

† *P* = 0.33 for the difference between boys and girls.

**TABLE 4:** HBV infection amongst HIV-positive and HIV-negative infants stratified by age, KwaZulu-Natal, South Africa (July 2011 to December 2011).†

Age category‡	HBV-positive (%)	HBV-negative (%)
≤ 6 weeks	9 (10.5)	77 (89.5)
> 6 to ≤ 24 weeks	16 (11.4)	124 (88.6)
25 to ≤ 52 weeks	5 (10.6)	42 (89.4)
> 53 weeks to 18 months	0	10 (100)

HBV, hepatitis B virus.

† 39 samples were excluded from this analysis because exact age could not be verified.

‡ *P* = 0.73 The chi-squared test showed that there was no difference in HBV infection across all age categories.

## Discussion

From this cohort of samples referred to an academic laboratory for routine early-infant diagnosis of HIV, we found a 10% prevalence of HBV infection for a sub-population of infants of the KwaZulu-Natal province of South Africa. The World Health Organization (WHO) categorises sub-Saharan Africa as highly endemic for HBV, with a prevalence of more than 8%.^25^ Exposure to HBV in different regions of the African continent is variable, as evidenced by the prevalence of antibodies to the HBV core antibody (anti-HBc). Anti-HBc prevalence in western Africa has been reported to exceed 85%, whereas the prevalence in eastern Africa is reported to range from 65% to 85%, with rural areas showing a higher prevalence than urban areas.^26^ A systematic review of global epidemiology of HBV over a 27-year period (1980–2007) showed a decrease in HBV prevalence in southern Africa, from around 7% in 1990 to just under 4% in 2005.^2^

South Africa has also been shown to have a variable HBV seroprevalence between the different provinces, with rural South African regions being reported to have a higher prevalence than their urban counterparts.^27^ In the pre-immunisation era, hepatitis B surface antigenaemia was reported to range from 10% amongst urban children to 18.5% amongst rural children in KwaZulu-Natal.^13^ A retrospective database analysis conducted for a nine-year period (2002 to 2010) in a reference laboratory in KwaZulu-Natal estimated the overall seroprevalence of hepatitis B surface antigen in all age groups to be 12.05%.^27^ Our finding of a 10% prevalence in this infant cohort is clinically significant. Although previously HBV prevalence has been mostly reported using serological markers, the use of HBV DNA as a screening tool can identify even occult infections. Occult HBV infection is defined as the presence of HBV DNA without hepatitis B surface antigen outside the acute-phase window period.

Most HBV infections in endemic areas, including sub-Saharan Africa, are thought to be due to transmissions in the perinatal period and early childhood.^28,29^ The high prevalence of HBV infection amongst infants in this study affirmed this assertion. Studies on the natural progression of HBV infection have shown that perinatally acquired infections progress to chronic hepatitis B, which is worrying for vaccination efforts.^31^ Our study specifically considered HBV rates amongst HIV-positive and HIV-negative infants and therefore differs from previous studies conducted in the pre-HIV epidemic era.^4,13^ Further, in the HIV era, higher prevalences of HBV have been reported amongst HIV-positive adults^7,8,9,10^ owing to shared routes of transmission. However, the observed prevalence of HBV amongst HIV-positive infants in our study was not statistically significant compared with HIV-negative infants, suggesting that high HBV infection rates may be independent of HIV in infants. Similar findings have been reported by studies of pregnant women in South Africa.^11,30^

Analysis of data following age stratification showed an equivalent distribution of HBV infection, with almost 10% of infections observed amongst infants aged 6 weeks or younger, suggesting congenital infection in this sub-group. Routine screening of pregnant women for HBV infection is still not policy in the South African public health care sector. The recommended treatment for infants exposed to HBV *in utero* is the administration of the HBV vaccine and HBV-specific immunoglobulin at birth.^32^ The availability of HBV vaccine and HBV-specific immunoglobulin for passive active immunisation at birth remains a challenge in most South African obstetric units.^33^ The WHO recommends HBV vaccination within 24 hours of birth. Providing this birth dose is feasible within the South African immunisation schedule, as other vaccines are currently given at birth. We propose that the birth dose of HBV vaccination, which has been shown to reduce early perinatal transmission,^34^ should be considered as part of the expanded programme on immunisation in South Africa.

### Limitations of the study

Testing was conducted on residual samples that were referred for early-infant diagnosis of HIV, which may have created a bias towards infants who had been exposed to HIV *in utero*, impacting the generalizability of our findings to the broader population of all infants in KwaZulu-Natal province of South Africa. In addition, no follow-up was conducted to confirm chronic HBV infection. Finally, the sample size of our study was relatively small limiting the statistical power to detect differences between HIV-positive and HIV-negative infants.

### Conclusion

This study found a clinically significant prevalence of HBV infection (10%) amongst a sample of HIV-positive and HIV-negative infants in KwaZulu-Natal, South Africa. The difference in HBV prevalence between the two groups of infants was not statistically significant, suggesting that HBV infection amongst children in this region may be a problem independent of HIV.
